# From sink to source: Regional variation in U.S. forest carbon futures

**DOI:** 10.1038/srep16518

**Published:** 2015-11-12

**Authors:** David N. Wear, John W. Coulston

**Affiliations:** 1 United States Department of Agriculture Forest Service, PO Box 8008 North Carolina State University, Raleigh, NC 27695, USA; 2 United States Department of Agriculture Forest Service, 4700 Old Kingston Pike, Knoxville, TN 37919, USA

## Abstract

The sequestration of atmospheric carbon (C) in forests has partially offset C emissions in the United States (US) and might reduce overall costs of achieving emission targets, especially while transportation and energy sectors are transitioning to lower-carbon technologies. Using detailed forest inventory data for the conterminous US, we estimate forests’ current net sequestration of atmospheric C to be 173 Tg yr^−1^, offsetting 9.7% of C emissions from transportation and energy sources. Accounting for multiple driving variables, we project a gradual decline in the forest C emission sink over the next 25 years (to 112 Tg yr^−1^) with regional differences. Sequestration in eastern regions declines gradually while sequestration in the Rocky Mountain region declines rapidly and could become a source of atmospheric C due to disturbances such as fire and insect epidemics. C sequestration in the Pacific Coast region stabilizes as forests harvested in previous decades regrow. Scenarios simulating climate-induced productivity enhancement and afforestation policies increase sequestration rates, but would not fully offset declines from aging and forest disturbances. Separating C transfers associated with land use changes from sequestration clarifies forests’ role in reducing net emissions and demonstrates that retention of forest land is crucial for protecting or enhancing sink strength.

The sequestration of carbon (C) in forests has partially offset net C emissions in the United States (US) over the past two decades but terrestrial C sinks cannot expand indefinitely and are altered by numerous factors^1^. As signatories to the United Nations Framework Convention on Climate Change, the US develops annual estimates of all carbon (C) sources and sinks from 1990 to the present following prescribed Intergovernmental Panel on Climate Change (IPCC) good practice guidance^2^,^3^ referred to as the National Greenhouse Gas Inventory (NGHGI). The most recent NGHGI reports annual forest C accumulation (including both sequestration and land use transfers) in the US (including coastal Alaska and Caribbean territories) at 223 Tg yr^−1^, roughly 0.5% of the stored forest C. Future forest C sequestration could influence emission reduction targets for other sectors of the economy and influence the costs of achieving policy goals, especially in the short run while transportation and energy sectors are transitioning to lower-carbon technologies^3^,^4^. Projections of forest C are needed to shape national greenhouse gas emission commitments and help design policies to increase forest C sequestration.

We simulate futures for forest C and CO_2_ emission offsets for regions of the United States based on a bottom-up approach^5^ that accounts for the species and age class structure of current forests and dynamics that influence the rate at which C is accumulated or emitted^6^. Land use change, climate change, forest aging, and forest disturbance including cutting, fire, and insects/diseases simultaneously influence C storage and sequestration within a region’s forests^7^,^8^,^9^,^10^,^11^,^12^. Land use change transfers C into and out of the forest land use. Climate change defines complex and uncertain adjustments to net C accumulation in forests. Several studies suggest that atmospheric enrichment from CO_2_ and N could increase biomass growth by 0–2% annually^13^,^14^,^15^. Other climate change impacts including change in growing season length, water availability, and temperature interact with atmospheric changes to determine growth responses^16^,^17^. Forest growth rates peak and then slow as forests mature^9^ so forest aging is a significant driver of sequestration. Natural disturbances (e.g. fire, weather, insects and diseases) and human-mediated disturbances (e.g. forest cutting) can result in C emissions, alter accumulation rates, and modify the forest age structure^18^,^19^,^20^,^21^. We apply empirical methods to field-sampled data, collected using an equal probability repeated measure design across all states in the conterminous US to simulate dynamics of forest conditions from aging, harvesting, and disturbances based on observed conditions and changes. Land use change, productivity shifts due to climate change, and a policy alternative are addressed using scenarios. Previous studies address one or more of these forces^20^,^21^,^22^,^23^,^24^,^25^ but none have addressed the full set for the US.

## Results

**Figure 1:**
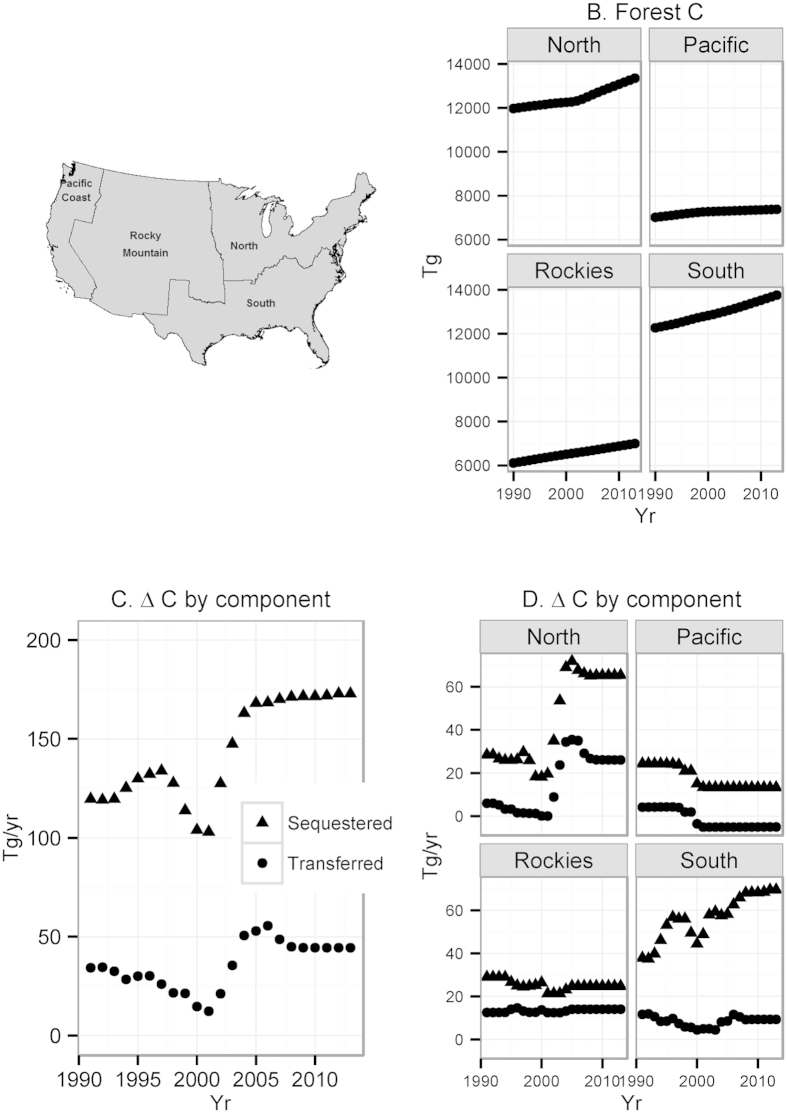
US forest C inventory dynamics by region and component. (**A**) Assessment regions. (**B**) Forest C inventory by region, 1990–2013. (**C**) Change in the US forest C inventory (1990–2013) decomposed into C transferred via land use change and the C sequestration (including disturbance related mortality and growth). (**D**) Change in forest C by region, 1990–2013, decomposed into C transferred via land use change and the C sequestration (including disturbance related mortality and growth). The map was created using R 3.0.3^©^ 2014 The R Foundation for Statistical Computing (http://www.R-project.org/)

The US NGHGI^3^,^26^ shows that forest C increased between 1990 and 2013 from 37 375 Tg to 41 498 Tg in the 48 conterminous US (we exclude Alaska and the Caribbean territories from our analysis—representing 3% of US forest C accumulation—due to data limitations). The recent rate of change (217 Tg yr^−1^ for 2013; Fig. 1C) reflects the net accumulation of forest area (some States have net forest losses while most have gains), forest growth, and the effects of forest disturbance. Using State level data, we decompose these changes into C transferred with land use changes and net C sequestered from the atmosphere by forests. Net forest area expansion transferred 44 Tg yr^−1^ into forest land use from other land use categories or about 20% of total forest C change (Fig. 1C). After accounting for forest C losses from cutting, land conversions, and disturbance and C accumulation from forest growth, the net forest C sequestration was 173 Tg yr^−1^, the equivalent of 634 Tg CO_2_.

The eastern US contained 65 percent of the forest C inventory in 2013 (33 percent in the South and 32 percent in the North; Fig. 1B). The Rocky Mountain and Pacific Coast regions in the West contained 17 and 18 percent respectively. In the Rockies, forest area expansion transferred 14 Tg yr^−1^into forests and C sequestration totaled 25 Tg yr^−1^ (Fig. 1C). In the Pacific Region, forest growth sequestered 13 Tg yr^−1^, while net losses of forest area resulted in a transfer of −5 Tg yr^−1^ from forests to other land uses. In the South, C sequestration dominated land use transfers (70 versus 9 Tg yr^−1^; Fig. 1D) while in the North sequestration and transfer were both large (65 and 26 Tg yr^−1^ respectively). Of the total C sequestration in 2013, 78% occurred in the East; 22% in the West. While forest C sequestration grew in the East between 1990 and 2013, it declined in the West—most of the increase in forest C accumulation between 2000 and 2010 results from forest area expansion in the East (Fig. 1D).

### Forest C projections

We projected forest C for twenty five years based on five scenarios (Table 1). Three address alternative land use outcomes including a Reference scenario. We then perturb the Reference scenario to allow for increased forest growth driven by climate factors (Enrichment scenario) and a policy case based on a program of afforestation (Policy scenario). Twenty-year forest area trends in the NGHGI show a slight but consistent net increase in forest area in the United States (705 thousand hectare per year between 2007 and 2012) while some projections^10^ suggest forest area peaking within two decades and then decreasing. For this twenty five year projection period, land use scenarios range from an extrapolation of the most recent decade’s rate of land use change to one which holds forest area constant. All scenarios apply recent historical (2002–2012) harvest rates for the projection period.

Our Reference scenario anticipates a peaking of forest area by 2032, applying historical change rates from 2013–2022, fifty percent of historical rates from 2023–2032, and no change from 2032–2037. An additional scenario holds forest area constant and another extrapolates the most recent area changes through the simulation period. The Enrichment scenario augments historical productivity relationships using an additive net productivity increase of 0.4%yr^−1^, in line with previous studies based on an inventory approach^13^,^14^ and could apply to any land use scenario. The Policy scenario increases forest area using different policy mechanisms in western and eastern regions. In the East, we simulate shifting agricultural land to forest, consistent with the federal Conservation Reserve Program (CRP), which allows landowners to receive rental payments for restoring environmentally sensitive agricultural land. Noting that ~2.3 million hectares left the CRP program between 2007 and 2010^27^, we simulate the effects of adding 2.0 million hectares of forest land in both the North and the South. In the West where public ownership dominate forests we assume that policy would focus on forest restoration and simulate the reforestation of forests identified as persistently non-stocked in the inventories; 1.1 and 2.6 million hectares in the Pacific and Rocky Mountain regions respectively.

For all scenarios we simulate change in forest C inventories using empirical population models applied to current inventories derived from detailed field-based forest plot records (see Methods). In western regions, a life-stage transition model is applied at the State level to C density estimates for eight carbon pools (down dead wood, forest floor, live trees above ground, live trees below ground, standing dead wood, soil organic C, understory vegetation above ground, and understory vegetation below ground) and disturbance rates derived from plot records. In eastern regions, a full set of remeasured forest plots provide estimates of C stock changes, transitions by age class, disturbance type and land use changes^1^.

**Figure 2:**
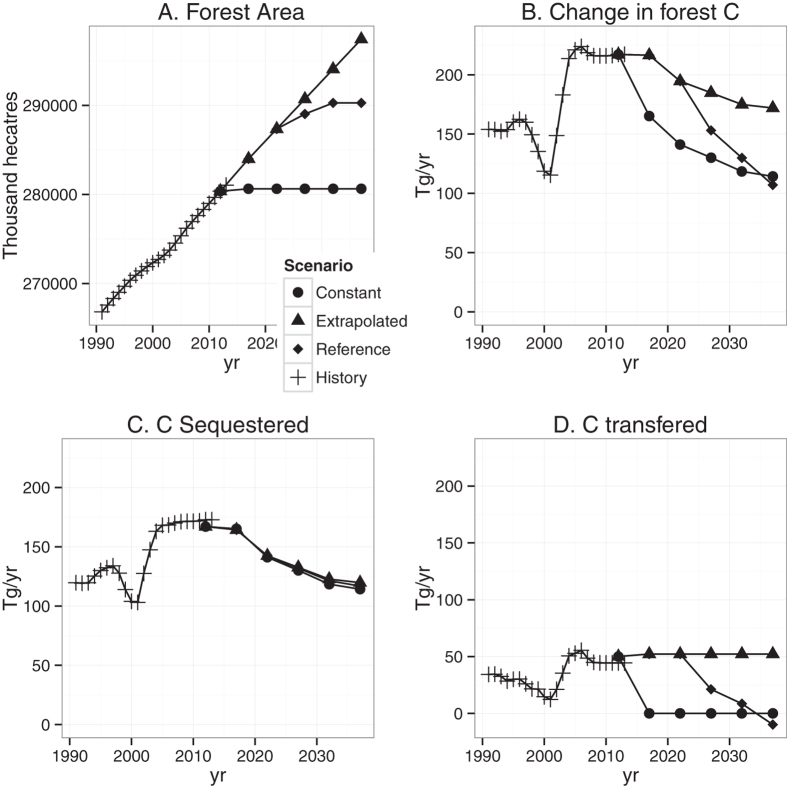
Projections of forest area and forest C for three scenarios. (**A**) US forest area (thousand hectares), (**B**) annual change in the forest C stock (*Tg yr*^−*1*^), (**C**) C sequestered by forests (*Tg yr*^−*1*^), and (**D**) C transferred to other land uses (*Tg yr*^−*1*^) for each of three land use scenarios: Constant forest area, Extrapolated forest area (based on previous 5 years change), and the Reference Scenario (transitions from extrapolated to constant at year 2032).

**Figure 3:**
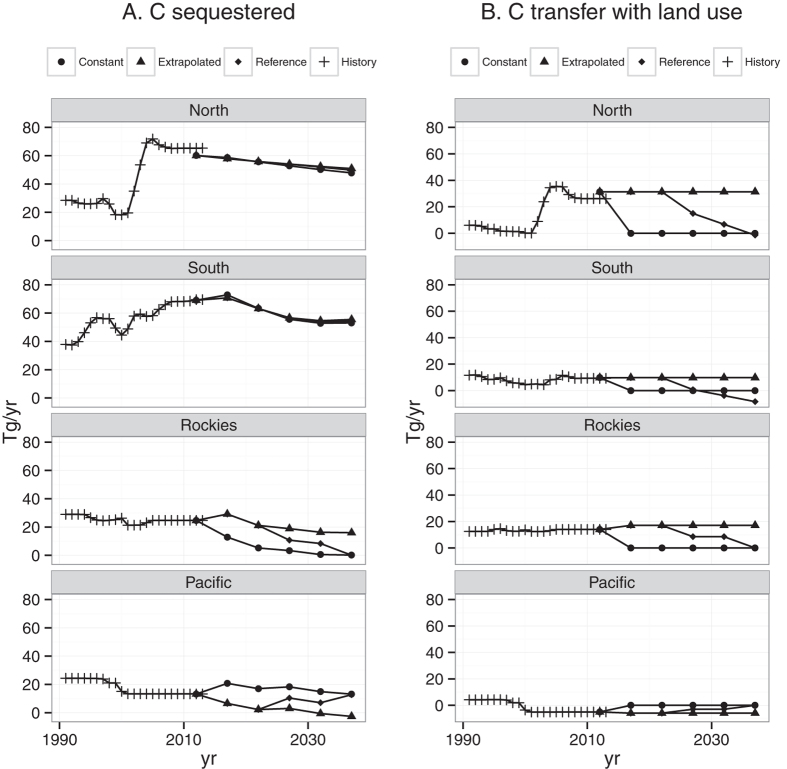
Regional breakdown of forest C projections by component for three scenarios. (**A**) C sequestered by forests (*Tg yr*^−*1*^) and (**B**) C transferred with land use change (*Tg yr*^−*1*^) for four regions of the United States for three land use scenarios: Constant forest area, Extrapolated forest area (based on previous 5 years change), and the Reference scenario (transitions from extrapolated to constant at year 2032).

Forest carbon stocks accumulate at decreasing rates in all regions for all land use scenarios. For the Reference scenario, US C sequestration rates fall by 35 percent from 173 Tg yr^−1^ in 2013 to 112 Tg yr^−1^ in 2037 (Fig. 2), while total C stock change decreases by 48% from 217 Tg yr^−1^ to 112 Tg yr^−1^. This highlights that changes in sequestration rates are likely to be less variable than changes in stocks that include land use transfers. For the Reference scenario, C sequestration in 2037 is 71% of 2013 levels in the North and 79% of 2013 levels in the South (Fig. 3). In the Pacific, sequestration fluctuates but remains near 2013 levels as net forest losses are eliminated by 2032. Sequestration in the Rocky Mountains declines to near zero by 2037.

**Figure 4:**
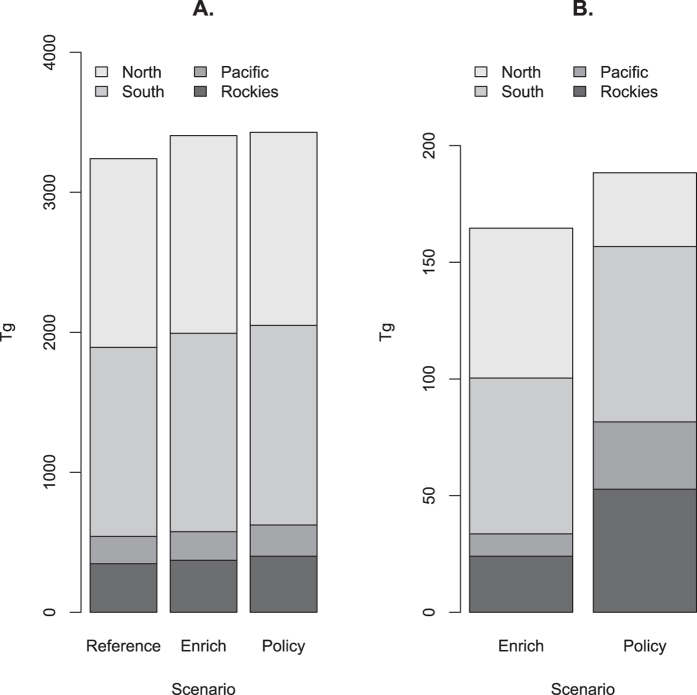
Alternative projections of forest C sequestered for enrichment and policy scenarios. (**A**) Forest C sequestered between 2013 and 2037 under three scenarios: 1) the Reference scenario, 2) the Reference scenario with a 0.4% yr^−1^ increase in forest growth, and 3) the reference scenario with policies for afforesting/reforesting land, and (**B**) Additional (beyond the Reference scenario) C sequestered between 2013 and 2037 by region for the Enrichment and Policy Scenarios.

The Constant forest area scenario isolates the influence of forest dynamics including disturbances, aging, and age-specific carbon densities (Fig. 3). In the South and the North, a very gradual slowing of sequestration reflects a strong growth response in early-stage forests (C growth peaks in the second decade) that offsets high rates of forest cutting. In the Rockies, a steep decline in sequestration indicates that forests in the Rockies are close to a balance between forest growth gains and disturbance losses. This region’s recent disturbance rates are high relative to long run historical values. In the Pacific, sequestration under constant forest area increases from 13 to 21 Tg yr^−1^ between 2012 and 2017 and stabilizes through 2037, reflecting high growth rates in forests harvested in the last half of the twentieth century. With the Reference scenario, US forests sequester 3,240 Tg (Fig. 4), an average of 129.6 Tg yr^−1^ between 2013 and 2037. The Enrichment scenario increases total sequestration by 5.1% over the Reference Scenario to 3,404 (136.2 Tg yr^−1^). 80% of the additional C under this scenario would be sequestered in the East (41% for the South and 39% for the North).

The policy scenario increases total sequestration by 5.8% over the Reference scenario to 3,428 Tg (137.1 Tg yr^−1^; Fig. 4). The C sequestered per unit area of afforestation is greatest in the South (1.48 t ha^−1^ yr^−1^) followed by the Pacific (1.10 t ha^−1^ yr^−1^), the Rockies (0.8 t C ha^−1^ yr^−1^), and the North (0.6 t C ha^−1^ yr^−1^). This ordering reflects the interaction of regional growth rates for young forests and disturbance rates.

## Discussion

Between 1992 and 2013, the pool of forest C in the US grew from 37 529 to 41 498 Tg (10.6 percent) with annual expansion of between 115 and 224 Tg. After accounting for C transfers associated with land use transitions, net C sequestration amounts to between 103 and 173 Tg yr^−1^, peaking in 2013. Policy makers interested in reducing net C emissions in the United States need information about (1) the likely path of forest C sequestration based on the variables influencing future sequestration rates, (2) the likelihood of diminished sequestration, and (3) policy options that might forestall diminishment or enhance rates of sequestration. Our projection scenarios were designed to provide insights into these questions at a useful scale for policy makers.

Our findings indicate a persistent but declining rate of forest C sequestration at the national level between 2013 and 2037, but projections vary by region. Over the 25 year projection period forest C sequestration in the US declines from 173 to 112 Tg yr^−1^ (averaging 129.6 Tg C yr^−1^) and generally supports others findings^20^,^25^. For example, these projections are consistent with the lower end of a range of projections described in the First Biennial Report of the US under the UN Framework Convention on Climate Change^28^. The largest projected change is the rapid loss of sequestration in the Rocky Mountain region, declining from + 25 to 0 Tg yr^−1^ where timber harvesting and growth rates are the lowest (by total and proportion) and aging and disturbance govern forest area change. Sequestration in the Pacific Region stabilizes (+~13 Tg yr^−1^) as historical declines in forest area dampen to zero. In the East, sequestration declines very gradually and represents an increasing share of the national total from 78% in 2013 to about 91% in 2037. The regions with the most timber harvesting (the South followed by the North) therefore provide the most stable future sequestration.

The forest component of the US NGHGI combines C sequestered by the atmosphere with net C transfers between nonforest land and forest land to define total change in forest C. IPCC good practice guidelines^2^ recommend the separation of land use transfers from the evaluation of sinks where possible and our analysis shows that isolating land use transfers avoids overstating the increase in forest C when land comes into the pool and the loss of forest C when land exits the pool. In the work presented here, forest sequestration (CO_2_ eq) was 9.7% of US CO_2_ emissions while net land use transfer of C to forest was 2.5% of CO_2_ emissions in 2012. While land transferred to a forest use will accumulate more forest C in the future, the carbon transfers among land uses are not actually removals from/losses to the atmosphere so combining the two metrics can obscure the actual contributions of forests.

Atmospheric CO_2_ and N enrichment have been reported to increase C sequestration in the range of 0–2% annually^12^,^13^. Our results suggest a 0.4% yr^−1^ increase in forest productivity would enhance sequestration by 5.1% over the 25 year simulation period after accounting for the interactions of regional sequestrations rates with forest age structures and disturbances. The specification (functional form) of growth enhancement is ambiguous, and affects estimates of impacts. Further, productivity enhancement would likely vary across the regions due to the confounding effects of climate and other edaphic factors. Our results show that the responses to productivity enhancement would be greatest in the eastern regions, where current growth rates are highest. However, this treatment of productivity leaves unaddressed potential negative effects of climate changes on growth, for example through increased frequency and severity of disturbances^15^.

Afforestation/reforestation activities in all regions yield additional C sequestration after accounting for growth and disturbance rates. The strongest marginal effects occur in the large timber producing regions of the South and the Pacific due to rapid growth in young forests. Economic factors such as strong timber markets or carbon credits that encourage retaining or expanding forest land can therefore provide meaningful enhancement of C sequestration. The policy scenario considered here, afforestation of 7.73 million hectares, appears a plausible goal given the range of recent forest conservation activities in the US and proposals by the USDA^29^, and yields an additional 188 Tg C over the 25 year simulation period.

Across all projection scenarios the forest carbon inventory in the US grows but at decreasing rates. Because scenarios apply historical disturbance and harvest rates, the declining sequestration rates reflect the dominant influence of forest aging coupled with land use change. Our reference scenario transitions from net forest gains to stable forest area, but net forest losses for the US have also been projected^10^ and would result in a more rapid deceleration of sequestration. In 2012, forest sequestration offset 9.7% of CO_2_ emissions. The projected changes in forest sequestration under the reference scenario implies that, without forest-based mitigation activities, emission offsets decline and emissions in other sectors would need to be reduced further to reach emissions targets (e.g., the US intends to reduce net emissions by 26–28% between 2005 and 2025^30^).

Enhanced productivity through climate changes and CO_2_ fertilization remains a key uncertainty. Productivity gains could lead to enhanced sequestration, especially in the East but climate-driven changes in disturbance frequency and disturbance intensity could also have negative consequences, especially in the West. The range of outcomes related to land use change scenarios shows land use change to be perhaps the most important uncertainty regarding the strength of the forest sink, especially in the near future. Research efforts should focus on monitoring for shifting productivity at the field inventory level and understanding the influence of changing disturbance regimes on post-disturbance forest conditions. Ongoing monitoring efforts should eventually allow for a clear identification of C dynamics for all land use change categories.

## Methods

We used inventory data as provided by the US NGHGI^3^ and the USDA Forest Service Forest Inventory and Analysis program^31^ to quantify and project C stock and C stock change for the conterminous US. Total carbon was used in this analysis and included the following pools: down dead wood, forest floor, live trees above ground, live trees below ground, standing dead wood, soil organic C, understory vegetation above ground, and understory vegetation below ground. While the NGHGI reports forest C accumulation for Alaska of approximately 6.5 Tg yr^−1^, limited inventory data do not support the projection modeling described herein.

### Data

Data from the USDA Forest Inventory and Analysis (FIA) program^31^ are the basis for this work and for the U.S. forest C inventory program. The FIA program relies on a rotating panel statistical design with a sampling intensity of one 674.5 m^2^ ground plot per 2 403 ha of land and water area. A five-panel design (20% of the field plots typically measured each year) is used in the eastern U.S. and a ten-panel design (10% of the field plots) is used in the western U.S. From the measurements taken on the field plots C values are predicted for eight pools (down dead wood, forest floor, live trees above ground, live trees below ground, standing dead wood, soil organic C, understory vegetation above ground, and understory vegetation ground) using the models described by USEPA^3^.

Total C stock change as reported in the EPA’s annual Greenhouse Gas Inventory is estimated from the FIA data described above. These estimates are developed as described by USEPA^3^ according to IPCC good practice guidelines^2^. Historical data for forest C and forest C change are available at the State level.

### Greenhouse gas inventory data analysis

The forest component of the land use sector is one element of the national Greenhouse Gas Inventory and accumulation in forests involves transfers between forests and other land uses. Area that is transitioned from a non-forest use to a forest use is included as an increase in the forest C pool while transition from forest to non-forest use is included as a loss from the forest pool. Accordingly, net change in forest C includes both C exchange with the atmosphere and transfers to or from other terrestrial C pools. Additionally, some harvested forest C may be transferred to durable forest product C pools and this is accounted for in a separate component of the NGHGI. We decompose the total change in the forest C pool into a land transfer component and a forest growth component. The latter provides an estimate of the net C exchange between forests and the atmosphere. The NGHGI database provides State-level estimates of net changes in forest area (ΔA), total change in forest C (ΔC), and carbon densities by pool, including the average density of soil C at age zero (C_soil_) We approximate the C transfer associated with net forest area change as ΔA• C_soil_, and the C sequestered as:


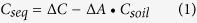


### Western projection model

In the western regions of the United States (Pacific and Rocky Mountain Regions in Fig. 1), where forest sampling is less intense and transition measures are not yet available, we model changes in forest C using inventory aggregates and a stage-class forest population model^32^,^1^. Consider the following general description of forest C inventory:


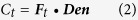


where 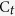 is total forest C at time t within the analysis area, **F** is a 1 × n vector of forest area by age class (n is the number of age classes and the n^th^ age class is a terminal age class, ideally defined where C reaches a stable maximum) and **Den** is an n × 1 vector of per unit area forest C densities arrayed by forest age class. Note that this can be generalized to account for multiple forest types or to distinguish among other relevant forest attributes (e.g., n = jk where j = the number of age classes and k = number of forest types). Inter-period forest C dynamics can be described by introduction of a transition matrix (**T**):


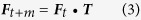


where **T** is an n × n matrix and each element 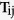 defines the proportion of forest area in class i transitioning to class j and m defines the time increment of the projection. The values of the elements of **T** depend on a number of factors, including forest disturbances such as harvests, fire, storms, and others, and the value of m, especially relative to the span of the age classes. For example, consider a case where we hold area fixed, allow for no mortality, define the time step m equivalent to the span of age classes, and define four age classes. **T** would be:


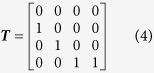


where all forest area progresses to the next age class and forests within the terminal age class are retained forever. With this version of **T**, after five time steps all forests would be in the terminal age class. Relaxing these assumptions changes the structure of **T**. If we account for disturbances including harvesting and fire that result in stand regeneration and allow for stochastic elements in forest aging:


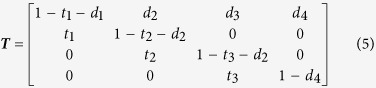


where 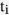 is the proportion of forest of age class i transitioning to age class i + 1, 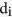 is the proportion of age class i that experiences a stand-replacing disturbance, and (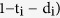 is the proportion retained within age class i.

Once **T** is specified, forest C at the end of the next period is defined as:


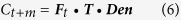


And change in C is defined as:


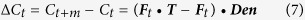


We can also incorporate land use change as a 1 × n vector **L** with positive entries indicating increased forest area and negative entries indicating loss of forest area.


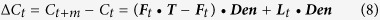



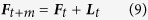


We model change in the West at the State level except in California, Oregon, and Washington where we separate the states into areas on the western and eastern sides of the Cascade mountain divide due to vast differences in forest productivity. For each State/substate we query the FIA inventory for all plot records to construct the C density vector (***Den***) by 5-year age classes from age 1–5 to greater than age 200. We also include an age class with recorded age of 0, which is largely composed of forests classified as non-stocked (where a land use change is not indicated but reforestation has yet to occur). For each State/substate we also define the age class distribution for the current forest area (**F**_1_). Non-stocked areas are treated separately in the simulations. For reference cases we assume that these forests persist in a non-stocked condition, but also explore scenarios where some of these areas would be restored to a productive condition. An average historical stand-replacing disturbance rate (d_i_) is defined by dividing the area of forests currently in the 1–5 year age class by total forest area (excluding non-stocked area) multiplied by the average transition rate (t). We apply this as a constant disturbance rate across age classes—our assumption then is that the recent disturbance pattern leading to forest replacement carries into the future. Note that d includes all events that reset the stand age including fire, weather, insects, and harvesting. We define an age transition rate (t_i_) of 0.85 for all age classes to complete the definition of the transition matrix ***T*** (equation 4).

Simulations proceed by applying equation 8, using ***Den, F***, and ***T*** matrices defined above and with areas of forest area change (***L***) defined by assumptions that vary by scenario. For net gains in forest area we assume that new forest is added to the 1–5 year age class; for losses we remove forests proportionately across all age classes. We calculate the C transfers associated with land use change assuming that the soil organic C component of the vector***Den*** transfers to/from the outgoing/incoming land use. Scenarios regarding future land use changes (incorporated in***L***), potential shifts in productivity (adjustments to the***Den*** vector), and forest restoration activities (through the ***T***matrix) drive a set of projections using these models. Separate simulations are constructed for each of the 18 State/subState units and results are summarized for Pacific and Rocky Mountain Regions. Model validation is conducted using the inverse of **T** 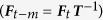 and historical land use change to backcast ***F***, C, and DC based on equations 6–8.

### Eastern projection model

Projection models for the eastern regions of the US (South and North regions in Fig. 1) utilize remeasured inventory plots. The preceding formulation of C inventory change is based on simulated forest type/age transitions and average C densities for given ages. A higher degree of specificity can be defined if we decompose the forest in a way that accounts for the effects of specific forest disturbances and other dynamics recorded for remeasured inventory plots. Consider the following modification to equation [8]:


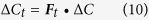


Where ∆C is total forest C change within the analysis area, **F** is as previously defined; **δC** is an n × 1 vector of per unit area forest C stock change per year arrayed by forest age class. Inter-period forest C dynamics are described as in equation [3] but the age transition matrix (**T**) is estimated from the observed data directly. Forest C change at the end of the next period is defined as:


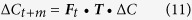


We incorporate land use change and cutting, fire, weather, and insects/diseases disturbances by generalizing equation [11] to account for the above change vectors and undisturbed forest remaining as undisturbed forest^1^:


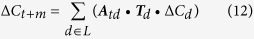


where **A** is the area by age class of each mutually exclusive land category in **L. L** is (FF, NFF, FNF, F_cut_, F_fire_, F_weather_, F_id_) where FF = undisturbed forest remaining as undisturbed forest, NFF = non-forest to forest conversion, FNF = forest to non-forest conversion, F_cut_ = cut forest remaining as forest, F_fire_ = forest remaining as forest disturbed by fire, F_weather_ = forest remaining as forest disturbed by weather, and F_id_ = forest remaining as forest disturbed by insects and diseases. When more than one disturbance occurs on a plot a dominant disturbance is assigned with cutting dominating when it occurs. Otherwise, the disturbance associated with the greatest tree mortality is assigned. In the case of land transfers (FNF and NFF), **T** is an n × n identity matrix and ***δC*** is a C stock transfer rate by age. Paired measurements for all plots in the inventory provide direct estimates of all elements of 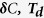, and ***A***_***td***_ matrices. Scenarios are implemented by adjusting ***A***_***td***_ for the NFF and FNF categories. Productivity shifts are implemented as adjustments to C stock change rates in 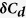.

### Disturbances

The effects of forest disturbances (e.g. cutting, fire, insects & diseases, weather) are included in this analysis as a “business as usual” case. Within the western modeling framework disturbances influence the Den vector and the T matrix. For example 8.2% of the forest area in the Pacific region had some amount of forest cutting (Table 2). Within the Rockies over 10% of the forest area had some degree of insect and disease damage. Within the eastern modeling framework the effects of disturbance are included more explicitly based on [11]. The primary disturbance in the east was cutting where 12.7% and 7% of the forest area in the South and North, respectively, experiences some-level of forest cutting. Forest cutting as described here includes not only clear-cut harvesting but also partial harvesting and thinning for forest management.

## Additional Information

**How to cite this article**: Wear, D. N. and Coulston, J. W. From sink to source: Regional variation in U.S. forest carbon futures. *Sci. Rep.* **5**, 16518; doi: 10.1038/srep16518 (2015).

## Figures and Tables

**Table 1:** Assumptions of the five scenarios evaluated including the projection of forest area change, policy, and productivity change.

Scenario Name	Forest Area Projection	Policy	Productivity
Extrapolated	Continue forest area change at historical rates (2012–2037)	Historical	Historical
Constant	Fixed forest area (2012–2037)		
Reference	Forest area change slows to zero at 2032	Afforestation/Restoration policy applied	
Policy			
Productivity		Historical	NPP incremented by 0.4% yr^−1^

**Table 2:** Average annual disturbance levels included in this analysis by region.

Region	Disturbance			
	Cutting	Fire	Insect & Disease	Weather
	Forest Area (%)			
North	7.0	0.5	4.4	3.3
South	12.7	3.4	1.3	2.1
Rockies	1.9	3.9	9.6	1.0
Pacific	8.2	3.7	10.3	1.9
